# Pentraxin 3 Promotes Glioblastoma Progression by Negative Regulating Cells Autophagy

**DOI:** 10.3389/fcell.2020.00795

**Published:** 2020-08-26

**Authors:** Zeyu Wang, Xing Wang, Nan Zhang, Hao Zhang, Ziyu Dai, Mingyu Zhang, Songshan Feng, Quan Cheng

**Affiliations:** ^1^Department of Neurosurgery, Xiangya Hospital, Central South University, Changsha, China; ^2^Department of Neurosurgery, West China Hospital, Sichuan University, Chengdu, China; ^3^One-Third Lab, College of Bioinformatics Science and Technology, Harbin Medical University, Harbin, China; ^4^Key Laboratory for Molecular Radiation Oncology of Hunan Province, Center for Molecular Medicine, Xiangya Hospital, Central South University, Changsha, China; ^5^Department of Clinical Pharmacology, Xiangya Hospital, Central South University, Changsha, China; ^6^National Clinical Research Center for Geriatric Disorders, Changsha, China

**Keywords:** pentraxin 3, autophagy, JUN, prognosis, glioblastoma

## Abstract

Glioblastoma is the most malignancy tumor generated from the central nervous system along with median survival time less than 14.6 months. Pentraxin 3 has been proved its association with patients’ poor survival outcome in various tumor. Recently, several studies revealed its association with glioblastoma progression but the mechanism is remained unknown. Autophagy is a programmed cells death and acts critical role in tumor progression. In this study, pentraxin 3 is recognized as prognostic prediction biomarker of glioblastoma and can promote glioblastoma progression through negative modulating tumor cells autophagy. Transcription factor JUN is assumed to participate in cells autophagy modulation by regulating pentraxin 3 expression. This work reveals novel mechanism of pentraxin 3 mediated glioblastoma progression. Furthermore, JUN is identified as potential transcription factor involves in pentraxin 3 mediated tumor cells autophagy.

## Introduction

Glioma is characterized as primary tumors that originate in brain parenchyma, which can be classified according to the type of glial cell involved in the tumor (Liu et al., 2016). World Health Organization characterizes glioma into four grades based on its malignancy. GBM, WHO grade IV, is the most vicious type with median survival time less than 15 months (Yang et al., 2011a; Hong et al., 2012; Ouyang et al., 2016). Current clinical treatment including maximum surgical resection followed by postoperative radio-therapy and concurrent chemo-therapy (Stupp et al., 2005; Gusyatiner and Hegi, 2018), but patients’ survival outcome remains unsatisfactory. Recently, several factors have been identified and be applied to predict survival outcome in clinical such as the subtype of GBM (Verhaak et al., 2010) and the status of IDH1 (Wang et al., 2013). On account of GBM heterogeneity, insight on potential prognostic prediction factors are urgent need.

Pentraxin 3, known as TSG-14, is an inflammatory molecule belongs to the pentraxin family and mainly secreted by inflammatory cells like dendritic cells and macrophages (Liu et al., 2011; Bonavita et al., 2015). Recently, PTX3 has been proved its role in tumor progression. For example, PTX3 affects tumor proliferation and apoptosis by interacting with the PI3K/AKT/mTOR signaling pathway in lung cancer (Ahmmed et al., 2019) and breast cancer (Thomas et al., 2017). PTX3 involves in the epithelial–mesenchymal transition in melanoma (Ronca et al., 2013) and breast cancer (Kampo et al., 2019). Notably, PTX3 can interact with the fibroblast growth factor-2/fibroblast growth factor receptor system to promote tumor progression (Bonavita et al., 2015; Ying et al., 2016; Giacomini et al., 2018). In glioma, previous study confirmed that decreased the expression of PTX3 impaired glioma cells proliferation and invasion ability (Tung et al., 2016). However, the role of PTX3 in GBM is poorly understood.

Cells autophagy is a programmed cell death and act as a response to unfavorable factors like hypoxia and nutrient deficiency (Stavoe and Holzbaur, 2019). Therefore, by activating cells autophagy can prevent tumor progression and increase tumor sensitivity to chemo- or radio-therapy (Xu et al., 2018; Perez-Hernandez et al., 2019). Previous studies proved PTX3 can affect cells autophagy but their relationship in GBM is unknown (Giorgi et al., 2015; Wu et al., 2015).

In this study, we analyzed the expression profile of PTX3, its ability to predict survival outcome and potential mechanisms in affecting GBM progression based on The Cancer Genome Atlas (TCGA) dataset. Results were verified in the Chinese Glioma Genome Atlas (CGGA) dataset. Then, we performed *in vitro* experiments to prove PTX3 affects tumor cells viability and autophagy. By integrating results from experiments *in vitro* and bioinformatics, we proved PTX3 negative modulates cells autophagy, and transcription factor JUN might regulate PTX3 expression.

## Materials and Methods

### Cell Culture and Transfection

Human GBM cells (U87-MG) are purchased from the Chinese Academy of Sciences. Glioma cells are maintained in DMEM medium with 10% fetal bovine serum and 1% penicillin–streptomycin, 5% CO_2_ and 37°C. Cells are randomly divided into different groups, the control group, the siRNA-NC (si-NC) group, the siRNA-PTX3 (si-PTX3) group, the overexpression JUN group and the overexpression JUN with siRNA-PTX3 group.

The siRNA of PTX3 (5′-GGTCAAAGCCACAGATGTA-3′) and JUN overexpression plasmid are obtained from RiboBio (Guangzhou, China). Five microliters of siRNA-PTX3 (or 2.5 μg overexpression JUN plasmid) and 5 μl lipofectamine (RiboBio, China) are added into 100 μl serum-free DMEM. Then, 1 ml DMEM is added and the mixed solution is incubated at 37°C for 6 h. The medium is discarded after 72 h and cells are washed by PBS twice for further experiment.

### Western-Blotting Assay

The expression level of beclin1, LC3B, PTX3, JUN, and β-actin were assessed by the Western-blotting assay. The bicinchoninic acid method (Thermo Fisher, Waltham, MA, United States) was performed as protocol to quantify the protein concentration. Based on the calculated protein concentration, protein samples were loaded and run on 10% SDS-PAGE gels at 75 V for 140 min. Then, proteins were electrotransferred onto PVDF membranes, and the membranes were incubated overnight at 4°C with 5% non-fat milk. Specific proteins were detected by anti-beclin1 (Proteintech Cat# 11306-1-AP, RRID: AB_2259061), anti-LC3B (Proteintech Cat# 18725-1-AP, RRID: AB_2137745), anti-PTX3 (Proteintech Cat# 13797-1-AP, RRID: AB_2165302), anti-JUN (Proteintech, Cat# 24909-1-AP, RRID:AB_2860574), and anti-β-actin (Proteintech Cat# 60008-1-Ig, RRID: AB_2289225). The second day, after washed three times with PBST for 15 min, the membranes were incubated with horseradish peroxidase-conjugated secondary antibodies (Proteintech Cat# SA00001-1, RRID: AB_2722565; Proteintech Cat# SA00001-2, RRID: AB_2722564) at room temperature for 90 min.

Autophagic flux assay adopted similar process as previous depicted. Cells were separated into five groups and was processed with autophagy inhibitor Bafilomycin A1 (Baf A1; Abcam). A: the control group without Baf A1; B: the si-PTX3 group without Baf A1; C: the si-PTX3 group with Baf A1; D: the control group with Baf A1; E: the si-NC group with Baf A1.

### CCK8 Assay

The logarithmic growth phase transfected tumor cells were obtained and digested for CCK8 assay. 5 × 10^3^ glioma cells and 100 μl of medium were placed into 96-well plates. The absorbance at 450 nm was measured per 24 h during the following 3 days.

### Colony Forming Assay

Cells were digested and plated in 6-well plates (300 cells per well) and cultured with 5% CO_2_ at 37°C for 2 weeks. The colonies were then fixed with 4% methanol (1 ml per well) for 15 min and stained with crystal violet for 30 min at room temperature. After photograph, discoloration was performed with 10% acetic acid, and cells were measured absorbance at 550 nm.

### Immunofluorescence Assay

Cells were fixed with 4% paraformaldehyde, then added with 0.3% triton at 37°C. After be blocked with 3% BSA for 60 min, cells were incubated with rabbit anti-LC3B (1:200, ab51520; Abcam) overnight at 4°C. At the second day, cells were incubated with fluorescein isothiocyanate (FITC)-conjugated secondary antibodies (green) at 37°C for 90 min, and stained with DAPI (blue) at 37°C for 10 min. Observation and photograph were conducted by confocal microscopy.

### Data Collection and Single-Cell Analysis

RNA-seq data of glioma and corresponding clinical information were acquired from the TCGA database^1^ and the CGGA database^2^. All data were transferred into TPM data for further analysis.

For single-cell analysis, three GBM samples from GSE139448 are processed and normalized by R package “Seurat,” “NormalizeData,” and “FindVariableGenes” (Wang et al., 2020). The GO analysis based on PTX3 expression is perform as mentioned above. Expression profile of PTX3 and JUN are plotted by the R package “vlnplot.” All data are obtained from online public database, and corresponding ethic statement can be found in their website.

### GO Analysis and Gene Set Enrichment Analyses (GSEA)

Genes with the adjusted *p*-value < 0.05 and the absolute FC larger than 2.0 were considered to be statistically significant. Gene Ontology (GO) analysis on the aberrantly expressed genes were determined by the GSVA analysis, and false discovery rate (FDR) < 0.05 were considered statistically significant. The GSEA analysis was conducted to illustrate the relationship between PTX3 expression and hallmark gene sets from the Molecular Signatures Database (MSigDB).

### Survival Analysis

Patients were subdivided into high and low groups according to median PTX3 expression. The overall survival (OS), progression free interval (PFI), and disease specific survival (DSS) rates of patients in low and high group were compared by the Kaplan–Meier method with log-rank test. ROC and AUC were performed to evaluate the prediction performance of PTX3 expression in various aspects, including 3,5-year OS, subtype of GBM (classical, mesenchymal, neural, proneural) and IDH status (wildtype, mutant).

### Mutation and Copy Number Variation Analysis

Single nucleotide polymorphisms (SNPs) and somatic CNVs were downloaded from the TCGA database. CNVs regions on chromosome associated with PTX3 expression were analyzed using GISTIC 2.0^3^. Venn diagram is generated by TBtools [Chengjie Chen, Rui Xia, Hao Chen & Yehua He. TBtools, a Toolkit for Biologists integrating various HTS-data handling tools with a user-friendly interface. Preprint at https://www.biorxiv.org/content/10.1101/289660v1 (2018)].

### Statistical Analyses

PTX3 expression profile difference with in WHO grades, GBM subtypes and treatment outcome were analyzed using Wilcoxon rank testing. Kaplan–Meier survival curves were generated and compared by using the log-rank test. The Pearson correlation was applied to evaluate the linear relationship between gene expression levels. Univariate and multivariate Cox regression analyses, and LASSO regression analyses were performed by R/BioConductor (version 3.6.2^4^).

Statistical analyses of the colony-forming assay and the CCK8 assay were carried out by GraphPad Prism (version 8.0^5^). Two-way ANOVA analysis followed with Tukey’s analysis for more than two groups. *P*-value < 0.05 was considered to be statistically significant.

## Results

### PTX3 Expression Is Elevated in Glioblastoma

**FIGURE 1:**
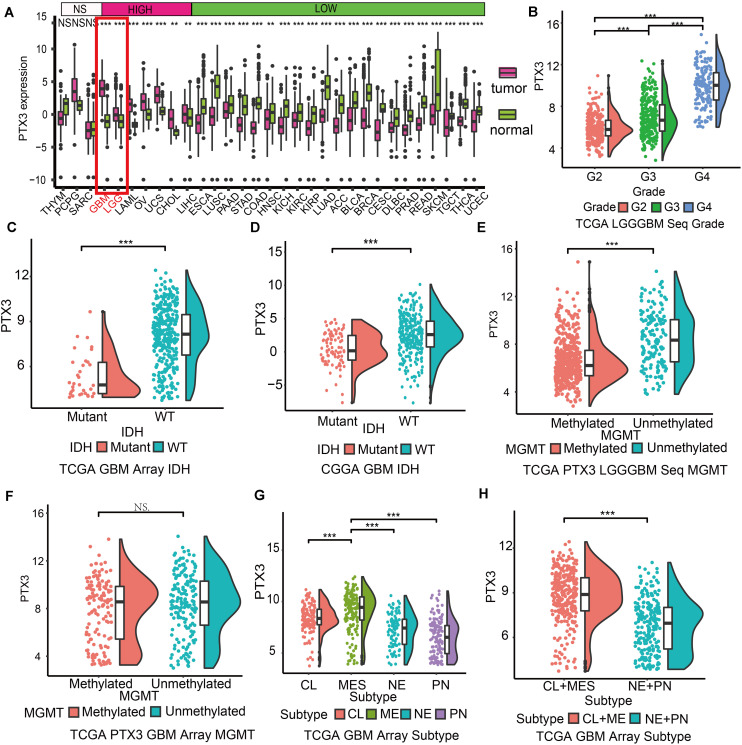
Expression profile of PTX3 in glioma. **(A)** Expression profile of PTX3 in different tumor cased on TCGA and normal tissue from the Getx database. **(B)** Sequence data of PTX3 mRNA levels in WHO II, III, and IV from the TCGA dataset. PTX3 expression is related to the status of IDH in GBM from the TCGA microarray dataset (**C**, *P*-value < 0.001) and the CGGA dataset (**D**, *P*-value < 0.001). PTX3 expression is significant elevated in MGMT unmethylated group than MGMT methylated group in glioma (**E**, *P* < 0.001) from the TCGA sequence dataset, while similar difference is not observed in GBM (**F**, array, *P* < 0.001) from the TCGA array dataset. **(G,H)** PTX3 expression profiles in GBM subtypes from the TCGA microarray dataset. MES, mesenchymal; PN, proneural; NE, neural; CL, classical. NS, no significantly statistical; *P < 0.05; **P < 0.01; ***P < 0.001.

PTX3 expression profiles of pan-cancer and normal tissue were obtained from the TCGA database and the Getx database. PTX3 expression in glioma is higher than normal tissue (*P* < 0.001; Figure 1A). In glioma, the expression of PTX3 is increased along with the tumor grade elevated (*P* < 0.001; Figure 1B). Based on treatment outcome after first course, PTX3 expression is significantly increased in patients with PD than other three groups (CR, PR, and SD) in glioma from the TCGA database (*P* < 0.001, Supplementary Figure S1A). Therefore, high PTX3 expression indicates worse survival outcome.

The IDH status serves as prognostic prediction biomarkers in clinical (Chen et al., 2019), and the MGMT status can predict tumor sensitivity to temozolomide (Hegi et al., 2005). In our work, PTX3 is enriched in IDH wildtype GBM (TCGA: *P* < 0.001, Figure 1C; CGGA: *P* < 0.001, Figure 1D), unmethylated glioma (*P* < 0.001, Figure 1E). But no significantly expression difference is observed in GBM cased on the MGMT status (*P* > 0.05, Figure 1F) in the TCGA dataset. As for GBM subtypes, mesenchymal GBM exhibits the worst survival outcome and highest PTX3 expression while proneural GBM to the opposite in the TCGA microarray database (Figures 1G,H and Supplementary Figure S1B). Therefore, high PTX3 is associated with aggressive glioma.

### PTX3 Acts as Prognostic Prediction Biomarker and Indicates Worse Survival Outcome

**FIGURE 2:**
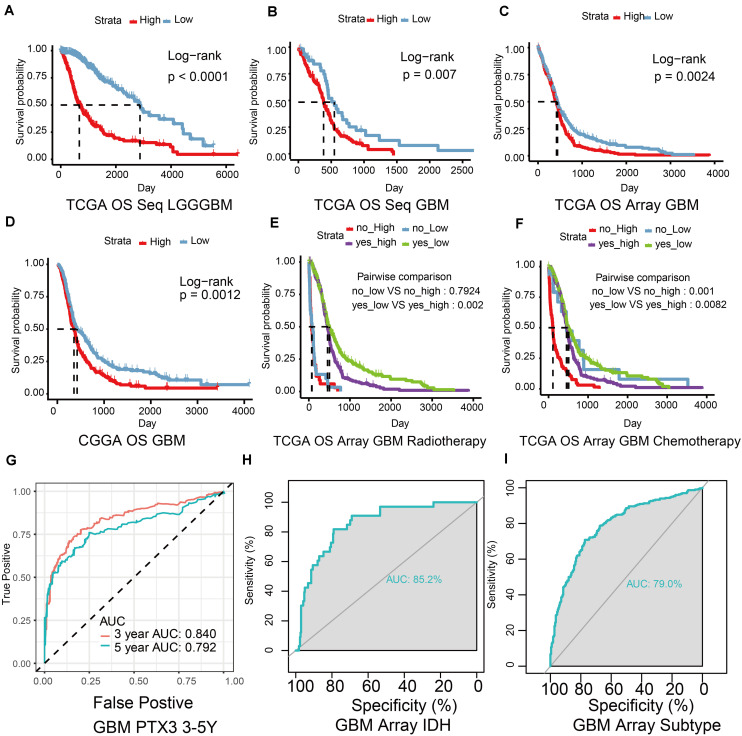
The ability of PTX3 to predict survival outcome. **(A)** The OS survival probability (*P*-value < 0.001) was calculated by the Kaplan–Meier survival analysis in glioma with high or low PTX3 expression from the TCGA sequence dataset. The OS in GBM from the TCGA sequence dataset (**B**, *P* = 0.007), the TCGA microarray database (**C**, *P* = 0.0024), the CGGA dataset (**D**, *P* = 0.0012). The survival outcome difference based on the expression of PTX3 in GBM based on the TCGA microarray database when taking radio- **(E)** or chemo-therapy **(F)** into consideration. **(G)** ROC curve of 3 (AUC is 0.840) and 5 years (AUC is 0.792) survive probability based on PTX3 expression in GBM from the TCGA sequence database. ROC curve of PTX3 expression based on the status of IDH (**H**, AUC = 0.852) and subtypes of GBM (**I**, AUC = 0.790).

Patients were subdivided into high or low risk group based on median PTX3 expression to analyze survival outcome difference. In the TCGA database, high PTX3 expression suggested worse survival outcome than low PTX3 expression in glioma (*P* < 0.001; Figure 2A). Similarly, low risk group manifested better survival outcome relative to high risk group in GBM in the TCGA sequence (*P* = 0.007; Figure 2B) and microarray (*P* = 0.0024; Figure 2C) database. Same result was also confirmed in the CGGA database (*P* = 0.0012; Figure 2D). The survival outcome of patients receiving radiotherapy in low PTX3 group was better than high PTX3 group in the TCGA microarray database (Accepted: *P* = 0.002; Not accepted: *P* = 0.7924; Figure 2E). In chemotherapy, high risk group exhibited worse survival outcome than low risk group (Accepted: *P* = 0.0082; Not accepted: *P* = 0.001; Figure 2F). The DSS (microarray: *P* = 0.0011, sequence: *P* = 0.041, Supplementary Figures S1C,D) and PFI (microarray: *P* < 0.0001, sequence: *P* = 0.006, Supplementary Figures S1E,F) analysis in GBM also suggested similar results.

ROC and AUC were calculated to reveal the prognostic prediction ability of PTX3. The 3, 5 years survival probability of PTX3 expression were calculated (3-years: AUC = 0.84, 5-years: AUC = 0.792, Figure 2G). The AUC calculated according to the status of IDH (AUC = 0.852, Figure 2H) and GBM subtypes (AUC = 0.79, Figure 2I) in the TCGA microarray database were also calculated. Same results were verified in the TCGA sequence dataset (IDH: AUC = 0.839, subtypes: AUC = 0.842, Supplementary Figures S1G,H). Univariate and multivariate Cox regression analysis were also to evaluate the prognostic prediction ability of PTX3 (Supplementary Tables S1, S2). Therefore, PTX3 promotes tumor progression and its expression can predict survival outcome.

### Biofunction Prediction of PTX3

**FIGURE 3:**
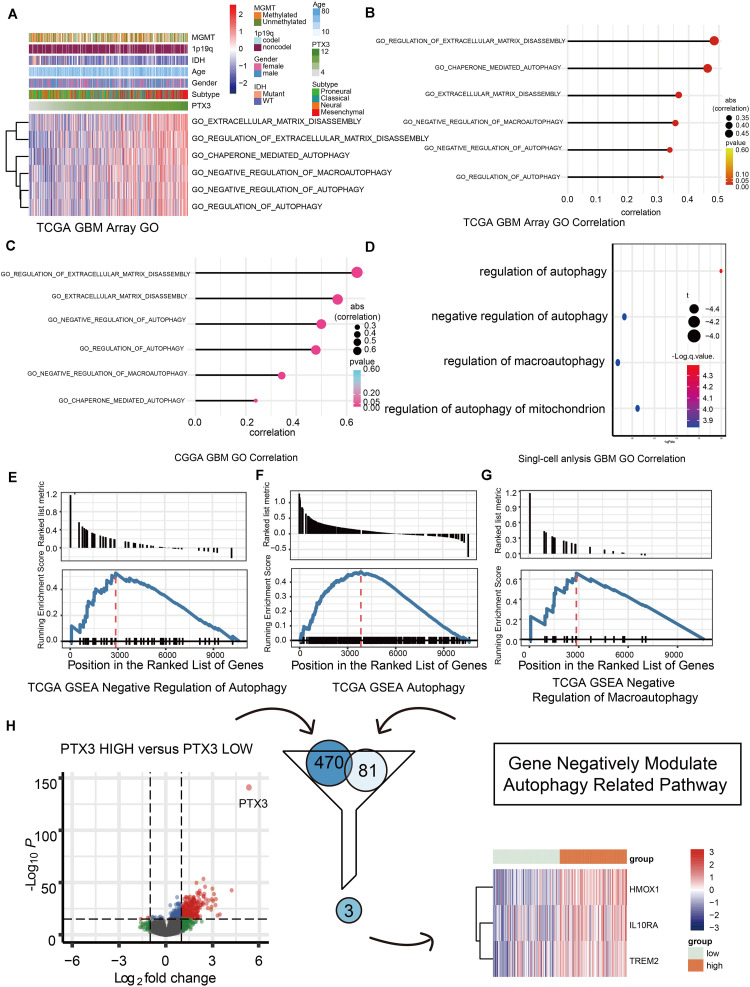
Biofunction prediction based on PTX3 expression. **(A)** The distributions of clinical features and gene set enrichment of different pathways according to PTX3 expression in TCGA. Correlation analysis was performed between PTX3 expression and GO pathways in GBM from the TCGA microarray dataset **(B)**, the CGGA dataset **(C)** and the single-cell analysis **(D)**. GSEA plots for enrichment of negative regulation of autophagy **(E)**, autophagy **(F)** and negative regulation of macroautophagy **(G)** in the TCGA dataset. **(H)** Three genes, HMOX1, IL10RA, and TREM2, are obtained by intersecting DEGs and genes involve in negative modulating autophagy.

Next, we predicted the potential biological functions of PTX3 by conducting the GO analysis (Figures 3A–C), the single-cell analysis (Figure 3D), and the GSEA analysis (Figures 3E–G and Supplementary Figure S1I). Results suggested that PTX3 involve in negative modulating cells autophagy and extracellular matrix disassembly. Therefore, PTX3 might promote tumor progression by inhibiting tumor cells autophagy. In order to explicit the association between PTX3 and genes involved in negative modulating autophagy pathway, we first identified differential expression genes (DEGs) between high and low PTX3 expression group. Then genes related to negative modulating autophagy pathway were obtained from the MSigDB^6^. Three genes, HMOX1, IL10RA, and TREM2, were identified by intersecting DEGs and autophagy related genes (Figure 3H). The correlation coefficient was also calculated (Supplementary Figure S2).

### PTX3 Affects Tumor Cell Viability by Negative Modulating Cell Autophagy

**FIGURE 4:**
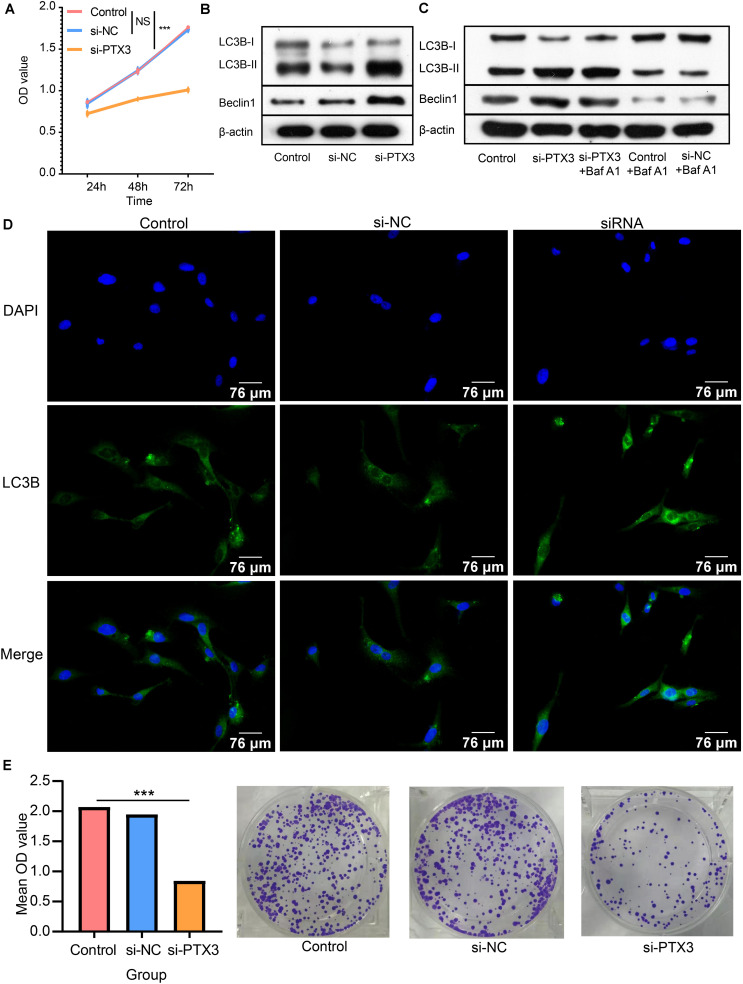
PTX3 affects cells viability and negatively modulate cell autophagy. **(A)** Cells proliferation ability is evaluated by CCK8 assay at 24 h, 48 h, and 72 h after interfering the expression of PTX3. **(B)** Western bolt illustrated autophagy related proteins, beclin1 and LC3B, are upregulated when PTX3 expression is inhibited. **(C)** The autophagy flux assay suggests the transition from LC3B-I to LC3B-II is inhibited when silenced PTX3 expression. **(D)** U87 MG cells are stained with DAPI (blue) and LC3B (green). Images are captured by laser confocal microscope (×400). **(E)** The colony-forming assay supports cells viability is inhibited by silencing PTX3 expression. NS, no significantly statistical; *P < 0.05; **P < 0.01; ***P < 0.001.

We next prove PTX3 can affect GBM cells viability. The CCK8 assay indicates that cells proliferation is inhibited by silencing PTX3 expression (Figure 4A). The Western-blotting assay suggests the expression level of autophagy related proteins, beclin1 and LC3B, are elevated in the si-PTX3 group (Figure 4B). Notably, the expression of LC3B-II is higher in the si-PTX3 group relative to other groups indicating activated cells autophagy.

Next, the autophagic flux assay is performed to determine the source of LC3B-II. Increased LC3B-II expression in the si-PTX3 group cannot be reversed by adding autophagy inhibitor Baf A1 (Figure 4C). Therefore, PTX3 can negative modulate cells autophagy. The LC3B expression profile is also examined by immunofluorescence suggesting U87MG cells in the si-PTX3 group tends to accumulate more LC3B in cytoplasm (Figure 4D). The colony forming assay also suggests the viability of U87MG cells is inhibited when PTX3 expression is decreased (Figure 4E). Thus, PTX3 expression affects tumor cells viability by negative regulating cells autophagy.

### Expression Profile and Biofunction of Transcription Factor JUN

**FIGURE 5:**
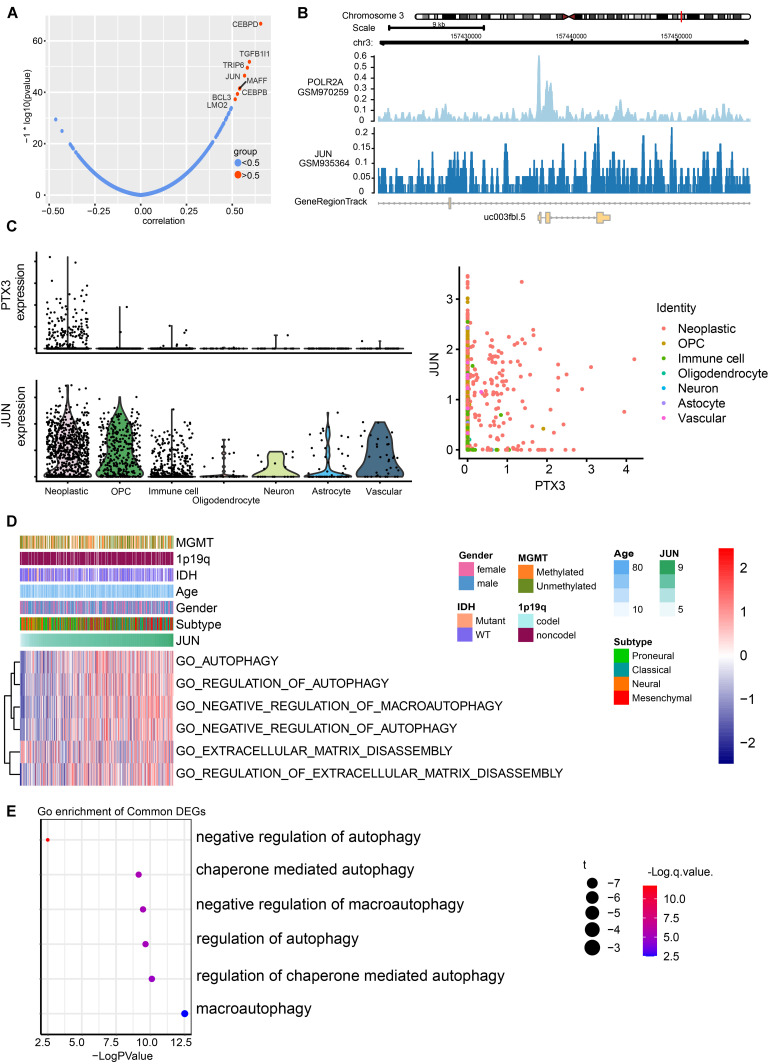
Prediction of transcription factors targets to PTX3 expression, and its potential pathways. **(A)** Correlation of potential transcription factors with PTX3. **(B)** The loci of JUN and POLR2A at chromosome 3 indicates JUN binds to the promoter of PTX3. **(C)** Expression profile of PTX3 and JUN based on single-cell analysis. **(D)** GO analyses based on JUN expression by using GSVA. **(E)** Correlation analysis between JUN and GO pathways in GBM from according to the single-cell analysis.

According to gene expression correlation, we identify positive correlation between JUN expression and PTX3 expression (Figure 5A and Supplementary Figure S3A). Likewise, JUN can predict survival outcome based on its expression (Supplementary Figures S3B,C). JUN can bind to chromosome 3 like polymerase (RNA) II (DNA directed) polypeptide A (regulator of message RNA synthesis) (Figure 5B). Previous study also proved that JUN binds to the promoter of PTX3 to regulate PTX3 expression (Chang et al., 2015). Next, we explore the distribution of PTX3 and JUN by single-cell analysis. JUN and PTX3 both enrich in GBM cells, except PTX3 also expresses in oligodendrocyte progenitor cells and immune cells (Figure 5C and Supplementary Figure S3D). Therefore, JUN might regulate PTX3 expression.

JUN expression can be applied to predict survival outcome, and high JUN expression indicates worse survival outcome in GBM (*P* = 001, Supplementary Figure S3B) and glioma (*P* = 0.026, Supplementary Figure S3C) based on the TCGA sequence database. The GO analysis based on JUN expression in TCGA database (Figure 5D and Supplementary Figure S3E) and single-cell analysis (Figure 5E) also support that high JUN expression is associated with negative modulating cells autophagy. JUN might affect tumor progression through regulating PTX3 expression.

### JUN Affect Glioblastoma Cells Proliferation, Viability and Autophagy

**FIGURE 6:**
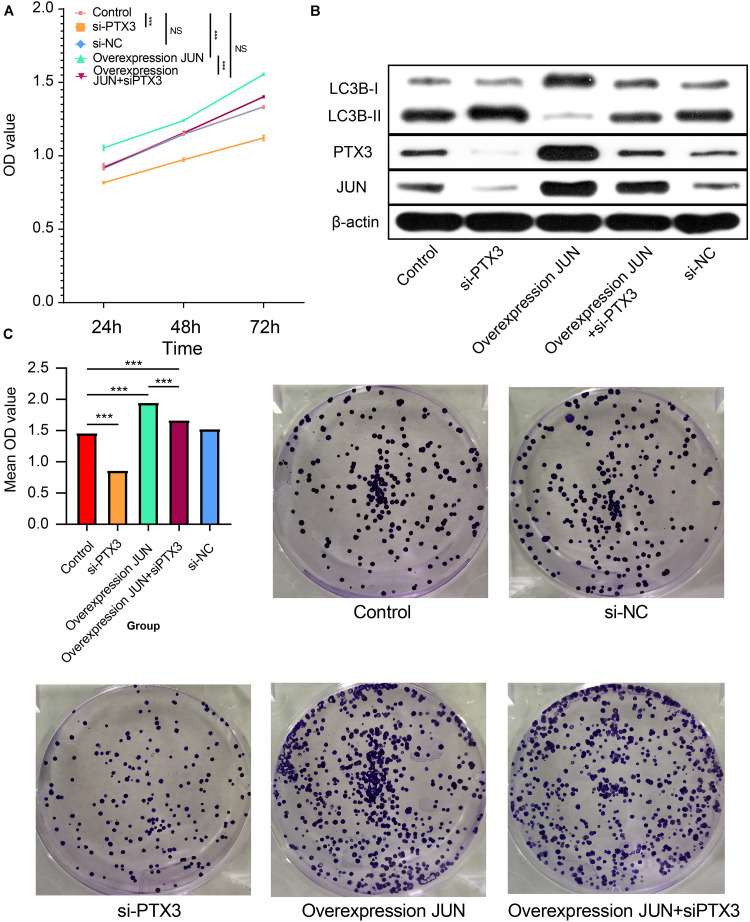
JUN involves in PTX3 mediated cell autophagy. **(A)** Cells proliferation was inhibited by silenced PTX3 expression. JUN overexpression promoted tumor cells proliferation, but it can be reversed by silenced PTX3 expression. **(B)** Western bolt suggested that JUN overexpression inhibited the LC3B-I to LC3B-II transition along with increased PTX3 expression. By simultaneously silenced PTX3 expression can restore that transition. **(C)** JUN overexpression can activate cells viability. By silencing PTX3 expression simultaneously can inhibit cells viability. NS, no significantly statistical; *P < 0.05; **P < 0.01; ***P < 0.001.

The CCK8 assay indicate that increased JUN expression can promote tumor cells proliferation, but by silencing PTX3 expression can reverse that process (Figure 6A). Next, the Western-blotting assay is performed to examine relationship between JUN, PTX3 and autophagy related proteins (Figure 6B). JUN can significantly increase PTX3 expression relative to the control group. In the meantime, less LC3B-I is transferred into LC3B-II indicating cells autophagy is inhibited. Cells autophagy is re-activated when PTX3 expression is inhibited. The colony forming assay also supported JUN can affect tumor viability through affecting PTX3 expression (Figure 6C). Therefore, JUN can affect U87MG cells proliferation, viability and autophagy.

### Epigenetic Changes and PTX3 Expression

**FIGURE 7:**
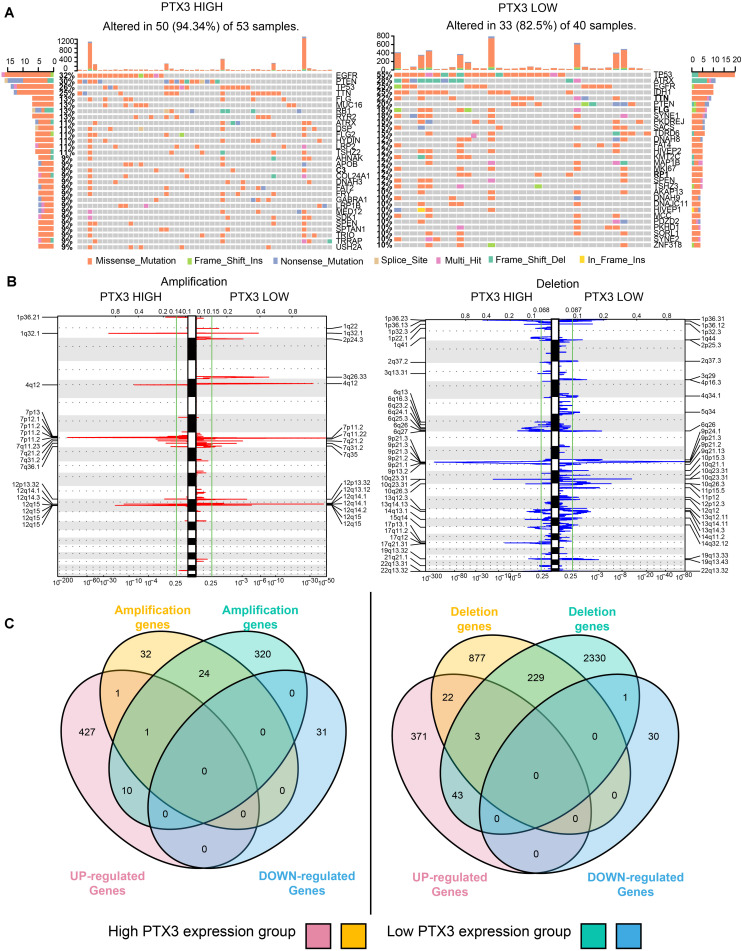
PTX3 high or low expression is associated with genomic alterations. **(A)** SNPs based on high and low PTX3 expression in GBM. **(B)** Frequency of chromosomal deletion (blue) or amplification (red) generated by GISTIC2.0 analysis and stratified by PTX3 expression. **(C)** Venn diagrams demonstrating the overlap regions of gene which is significant amplification (left) or deletion (right) in either PTX3 high or low expression, as well as the intersection with differential gene profiles.

Epigenetic alternation between high and low PTX3 expression in GBM is explored. SNPs suggested high gene mutation burdens are detected in high (50 of 53 samples) and low (33 of 40 samples) PTX3 expression group, including EGFR, PTEN, TP53, TTN, ATRX, IDH1 (frequency > 20%). Notably, EGFR (high: 32% vs. low: 25%) and PTEN (high: 30% vs. low: 20%) mutation are mainly enriched in high PTX3 group while ATRX (high: 11% vs. low: 28%), TP53 (high: 26% vs. low: 55%) and IDH1 (high: < 10% vs. low: 25%) mutation are enriched in low PTX3 group (Figure 7A).

GISTIC 2.0 is used to contrast the amplification and deletion region difference on chromosome based on PTX3 expression (Figure 7B). Amplification regions such as 1q32.1 (MDM4), 7q11.2 (EGFR, SEC61G), 12q15 (CPM), 12q14.1 (CDK4), and 4q12 (PDGFRA) are preferential discovered in high PTX3 expression group. Deletion regions such as 9p21.3 (CDKN2A), 1p36.23 (ERRFI1), 10q23.31 (PTEN), 9p21.3 (ELAVL2), and 17q11.2 (NF1) are enriched in high PTX3 expression group. In low PTX3 expression group, amplification region like 7q11.2 (SEC61G), 12q14.1 (CDK4), 4q12 (PDGFRA) and deletion regions like 9p21.3 (CDKN2A), 10q26.3 (PTEN), 10q26.3 (CYP2E1) are identified. To further illustrated DEGs is caused by CNVs or other regulating pathway, we identify amplification and deletion genes. The overlapping genes of CNVs and DEGs are obtained (Figure 7C and Supplementary Table S3). As noticed, few DEGs can be resulted from CNVs. Therefore, complicated regulatory network extensively exists in high and low PTX3 expression group.

## Discussion

Previous studies confirmed that elevated PTX3 level in tumor tissue as a biomarker of poor survival outcome (Locatelli et al., 2013; Tarassishin et al., 2014). In this work, we also prove PTX3 expression is associated with aggressive type of glioma. High PTX3 expression indicates worse survival outcome. By silencing PTX3 expression can impair tumor cells colony-forming and proliferation ability *in vitro*. Thus, PTX3 acts as a prognostic prediction biomarker of glioma.

**FIGURE 8:**
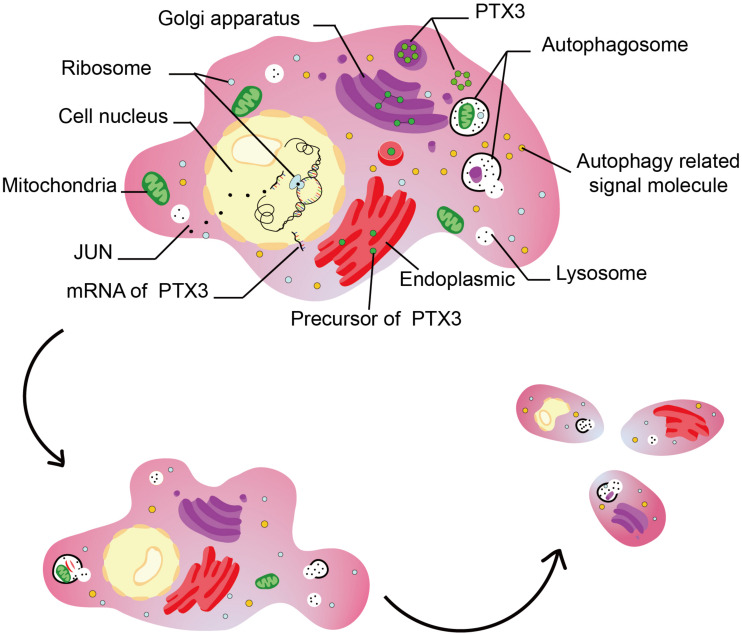
This figure illustrated the hypothesis that transcription factor regulates cell autophagy by modulating PTX3 expression.

Autophagy is a manner of programmed cell death, and highly conserved from microzyme to vertebrate mammal evolutionarily (White, 2015; Onorati et al., 2018). Autophagy deficiency triggers tumorigenesis by inducing oxidative stress, activation of the DNA damage response, and genome instability (Karantza-Wadsworth et al., 2007; Mathew et al., 2007, 2009). On the other hand, autophagy overactivation also promotes tumor growth, invasion, and metastasis (Guo et al., 2011; Lock et al., 2011, 2014; Yang et al., 2011b). Notably, autophagy is associated with tumor sensitivity to temozolomide (Yan et al., 2016; Xu et al., 2018). Therefore, dysregulated autophagy affects tumor progression. PTX3 has been confirmed relate to tumor migration, growth, sensitivity to radiotherapy (Bedini et al., 2018; Ahmmed et al., 2019; Rathore et al., 2019), but its relationship with autophagy in GBM is remained unclear. In our work, the GO and GSEA analysis suggest PTX3 negative regulating cells autophagy. *In vitro* experiment, we confirm that PTX3 negative mediates cell autophagy and promotes GBM cell proliferation. Notably, research reported that inhibited PTX3 expression significantly decreased tumor size on GBM xenografts (Tung et al., 2016). Therefore, PTX3 promotes tumor progression through negative regulating cells autophagy (Figure 8).

PTX3 was proved as an inflammatory factor belonging to the pentraxin family at the beginning (Bonavita et al., 2015). Its expression also enriches in immune cells based on the single-cell analysis. Therefore, PTX3 might able to affect tumor immune landscape (Netti et al., 2020). Tumor can be labeled as ‘hot’ or ‘cold’ according to their response to immunotherapy, and immunocytes infiltration degree decides tumor sensitivity to immunotherapy (Doni et al., 2019; Tomaszewski et al., 2019). Previous study proved PTX3 deficiency tumor manifested high macrophage infiltration, more cytokine production and high complement activation (Bonavita et al., 2015). However, the association between PTX3 and immunocytes is unclear and requires more researches.

JUN oncogene, also known as c-Jun or AP-1, belongs to the Jun family and encodes the component of the activator protein-1 complex (Meng and Xia, 2011). Previous study has already confirmed that JUN can bind to PTX3 promoter to regulate its expression (Chang et al., 2015). Other studies illustrated JUN serves as critical role in tumor progression. For example, the MAPK/JNK pathway can regulate cells autophagy, and c-jun is recognized as one of its downstream target (Zhou et al., 2015). The PI3K/Art pathway and the NF-κB pathway activation can initiate JUN expression in head and neck cancer (Chang et al., 2015). Factors like astrocyte elevated gene 1 (Liu et al., 2017), microRNA-4476 (Lin et al., 2020), 3-phosphoinositide dependent protein kinase 1 (Luo et al., 2018) can also activate c-jun expression. Therefore, JUN participates in cells autophagy regulation by affecting PTX3 expression.

Single nucleotide polymorphisms suggests mutation ratio of EGFR and PTEN are higher in high PTX3 expression group relative to low PTX3 expression group, while IDH1, ATRX and TP53 mutation are enriched in low PTX3 expression group. High EGFR and PTEN mutation are common in GBM, and actively participate in promoting tumor progression (Brennan et al., 2013; Han et al., 2016). TP53 is recognized as tumor suppressor and able to induce tumorigenesis (Wang et al., 2014). IDH1 and ATRX mutation have been confirmed as biomarker indicating better survival outcome in clinical (Wang et al., 2013; Aquilanti et al., 2018). Therefore, SNPs supported low PTX3 expression group indicates better survival outcome relative to high PTX3 expression group. CNVs indicates EGFR is amplificated in high PTX3 expression group while deletion regions like ERRFI1 and NF1 are mainly observed in high PTX3 expression group. Previous studies identified high EGFR expression promote tumor progression (Hatanpaa et al., 2010), and high ERRFI1 (Duncan et al., 2010) expression can slow tumor progression. In general, PTX3 is a prognostic prediction biomarker in GBM, and PTX3 promote GBM progression through negative modulating cells autophagy.

## Data Availability Statement

Publicly available datasets were analyzed in this study. This data can be found here: https://www.cancer.gov/about-nci/organization/ccg/research/structural-genomics/tcga; http://www.cgga.org.cn/; https://commonfund.nih.gov/GTEx/data. Experiment data can be obtained by contacting the corresponding author.

## Author Contributions

ZW, XW, and NZ prepared the manuscript, analyzed the data, and performed the experiments. HZ and ZD analyzed the data. MZ modified the manuscript. SF and QC designed the project and finally approved the manuscript to publish. All authors contributed to the article and approved the submitted version.

## Conflict of Interest

The authors declare that the research was conducted in the absence of any commercial or financial relationships that could be construed as a potential conflict of interest.

## Footnotes

1 http://www.tcga.org/

2 http://www.cgga.org.cn/

3 https://gatkforums.broadinstitute.org

4 https://www.r-project.org/

5 https://www.graphpad.com/scientific-software/prism/

6 http://www.gsea-msigdb.org/gsea/msigdb/cards/GO_NEGATIVE_REGULATION_OF_AUTOPHAGY

## Supplementary Material

FIGURE S1 Supplementary information about PTX3.

FIGURE S2 Correlation between PTX3 and three autophagy related genes.

FIGURE S3 Correlation analysis between JUN and PTX3 in GBM.

TABLE S1 Univariate Cox regression.

TABLE S2 Multivariate Cox regression.

TABLE S3 Gene list of Venn diagram.

## Abbreviations

AUC: the area under the curve
CGGA: The Chinese Glioma Genome Atlas dataset
CL: classic
CNV: copy number variation
CR: complete remission/response
DEGs: differentially expressed genes
EGFR: epidermal growth factor receptor
GBM: glioblastoma
LGG: lower grade glioma
MES: mesenchymal
NE: neural
PD: progressive disease
PN: proneural
PR: partial remission/response
PTEN: phosphatase and tensin homolog
PTX3: pentraxin 3
ROC: receiver operating characteristic
SD: stable disease
TCGA: The Cancer Genome Atlas dataset
TF: transcription factor
WHO: World Health Organization.
