# Supplementary material for: Pentraxin 3 Promotes Glioblastoma Progression by Negative Regulating Cells Autophagy
Source: Front Cell Dev Biol. 2020 Aug 26;8:795. doi: 10.3389/fcell.2020.00795 (PMC7479068; doi:10.3389/fcell.2020.00795)
Supplement: FIGURE S1 — Supplementary information about PTX3. [file Data_Sheet_1.pdf]

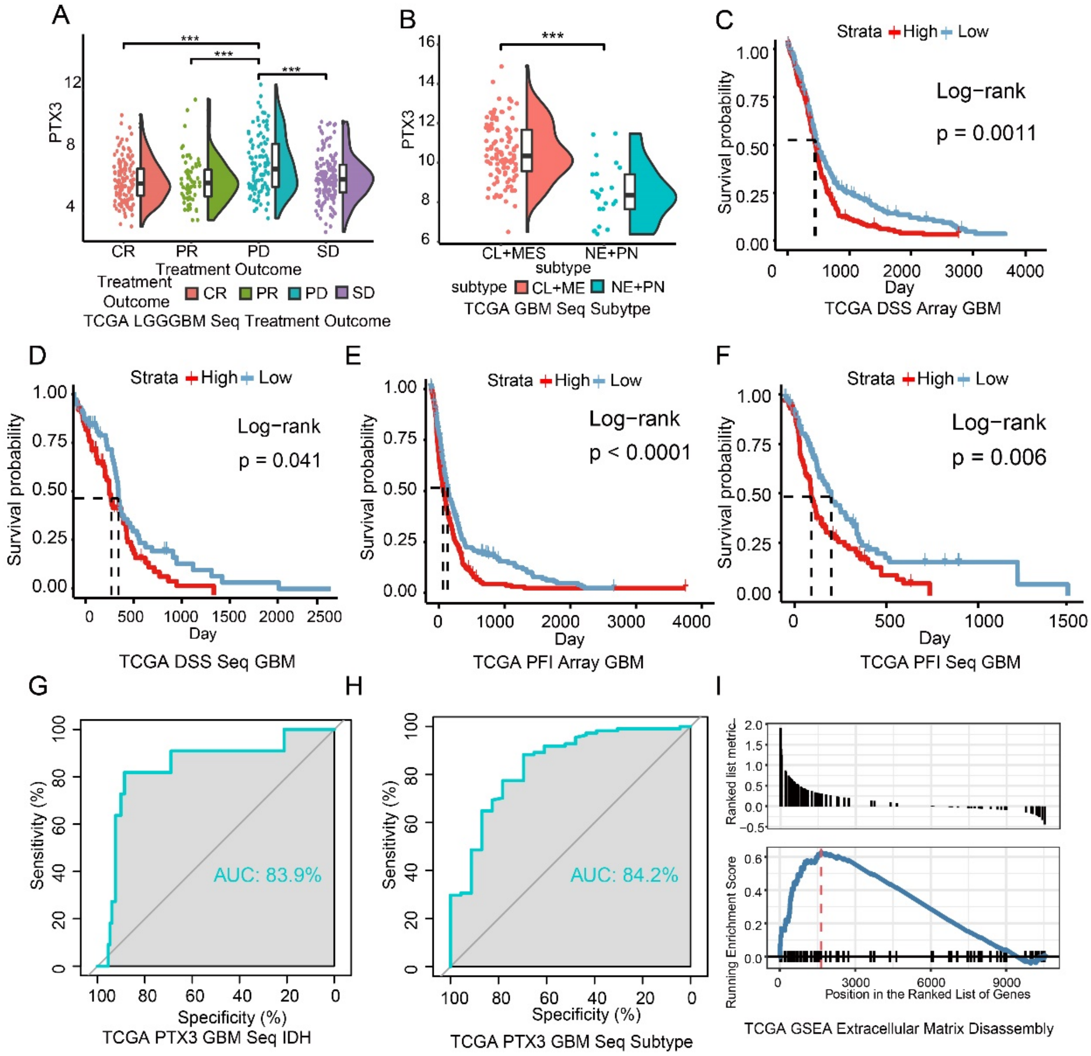

Supplementary Figure 1 | Supplementary information about PTX3. (A) PTX3 expression profiles in different treatment outcome after the first course in glioma based on TCGA sequence dataset. (B) Expression profile in GBM subtypes in the TCGA sequence database. The DSS (Array: P = 0.0011, C; Sequence: P = 0.041, D) and PFI (Array: P < 0.0001, E; Sequence: P = 0.006, F) survival analysis based on PTX3 expression in the TCGA database. (G-H) Receiver operating characteristic (ROC) curve based on the status of IDH (AUC = 0.839) and GBM subtypes (AUC = 0.842) in the TCGA sequence dataset. (I) The GSEA analysis suggested extracellular matrix disassembly as a potential biofunction of PTX3.

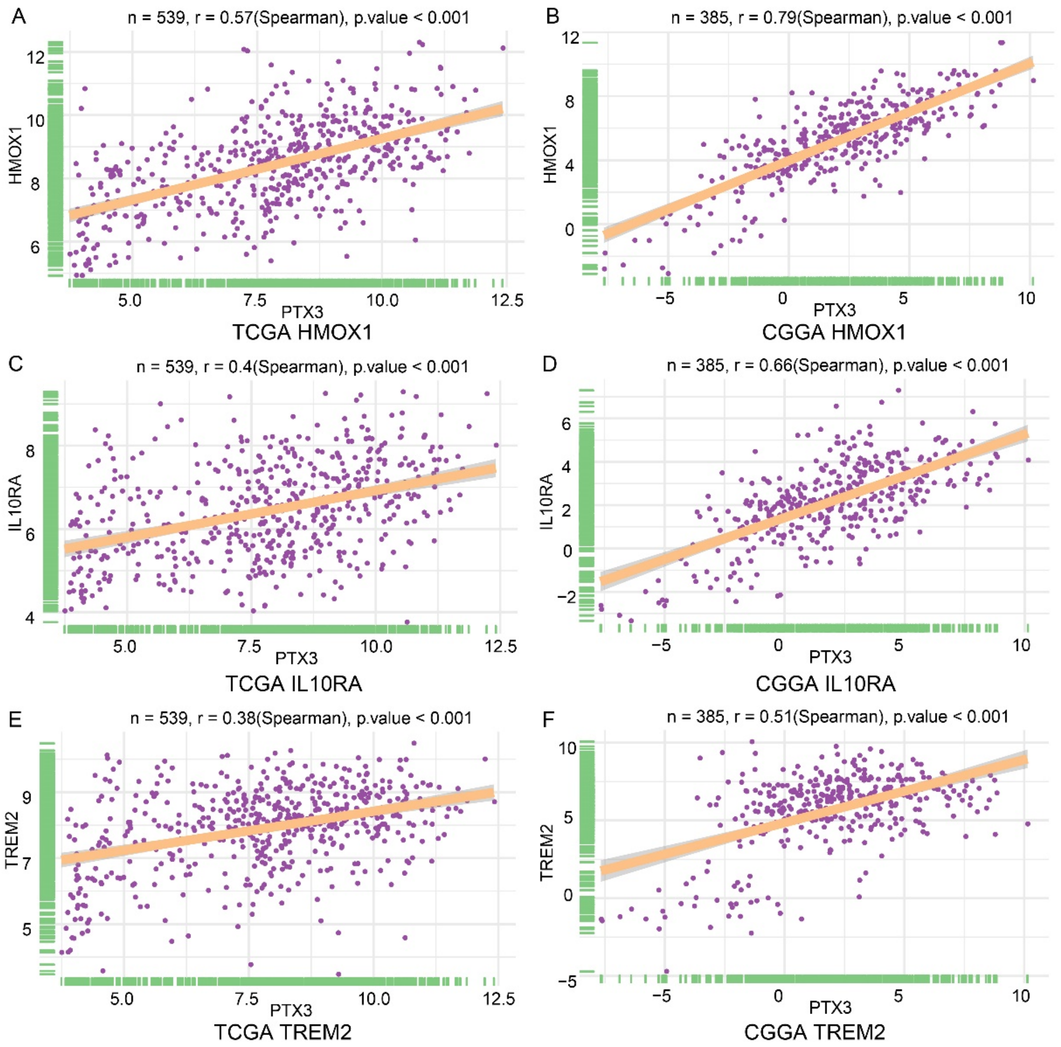

Supplementary Figure 2 | Correlation between PTX3 and three autophagy related genes. Correlation between PTX3 and three autophagy relate genes, HMOX1 (A, B), IL10RA (C, D) and TREM2 (E, F) from the TCGA and CGGA dataset. Correlation coefficient of HMOX1 (TCGA: r = 0.57; CGGA: r = 0.79), IL10RA (TCGA: r = 0.4; CGGA: r = 0.66) and TREM2 (TCGA: r = 0.38; CGGA: r = 0.51).

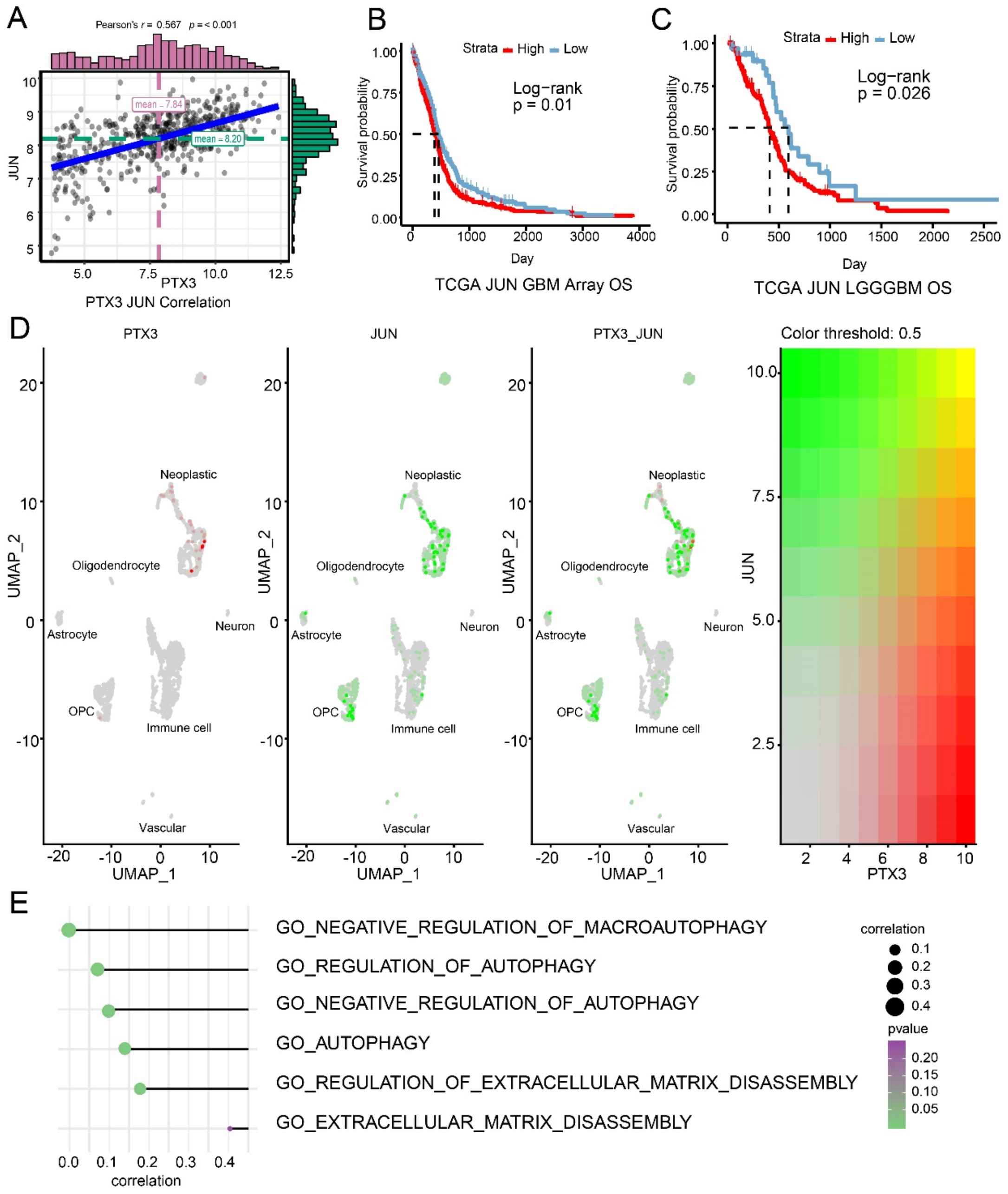

Supplementary Figure 3 | Correlation analysis between JUN and PTX3 in GBM. (A) Correlation analysis between JUN and PTX3 in GBM. The OS survival probability generated from the Kaplan-Meier survival analysis in GBM (B, P = 0.01) and glioma (C, P = 0.026) with high or low JUN expression in the TCGA microarray dataset. (D) Expression profile of JUN and PTX3 based on single-cell analysis. (E) Correlation of the GO analysis predicted pathways with JUN expression based on the TCGA database.

Database	TCGA				CGGA			
Factors	HR	HR.95L	HR.95H	P value	HR	HR.95L	HR.95H	P value
PTX3	1.112	1.06	1.19	< 0.01	1.07	1.02	1.11	< 0.01
Gender	1.17	0.92	1.49	0.19	0.98	0.77	1.24	0.86
Age	1.03	1.02	1.04	< 0.01	1.01	1.00	1.02	0.06
IDH	3.06	1.92	4.87	< 0.01	1.41	1.07	1.87	0.02
1p19q	4.29	1.39	17.71	0.04	1.96	1.12	3.43	0.02
MES	1.17	0.86	1.60	0.32	-	-	-	-
NE	1.07	0.76	1.51	0.71	-	-	-	-
PN	0.80	0.58	1.11	0.18	-	-	-	-

Supplementary Table 1 | Univariate Cox regression analysis.

Database	TCGA				CGGA			
Factors	HR	HR.95L	HR.95H	P value	HR	HR.95L	HR.95H	P value
PTX3	1.08	1.00	1.16	0.04	1.05	1.00	1.10	0.03
Male	1.28	1.00	1.64	< 0.05	0.95	0.75	1.21	0.70
Age	1.03	1.02	1.04	< 0.01	1.01	1.00	1.02	0.17
IDH-WT	1.92	1.09	3.37	0.02	1.07	0.78	1.49	0.69
Non-codel	4.06	0.89	18.50	0.07	1.65	0.92	2.97	0.10
MES	1.12	0.82	1.54	0.47	-	-	-	-
NE	1.29	0.90	1.85	0.16	-	-	-	-
PN	1.74	1.19	2.54	< 0.01	-	-	-	-

Supplementary Table 2 | Multivariate Cox regression analysis.

PTX3 expression	Overlap regions	Gene
HIGH	A-gene vs. up-gene	PDPN
HIGH	D-gene vs. up-gene	GATM, RYR3, SCG5, RRAS, ATF3, ITPKB, CNIH3, DPYD, F3, GBP1, GBP2, CYR61, ARHGAP29, TNFAIP3, FABP7, LAMA2, MOXD1, DSE, ATP1B2, ADAMTS1, C21orf7, SAMSN1
LOW	A-gene vs. up-gene	FLNC, GPR37, MEST, PODXL, PTN, ZYX, CLEC5A, DENND2A, TMEM176B, TMEM176A
LOW	D-gene vs. up-gene	SLC2A5, ADM, CD151, TPP1, SLC22A18, TRIM22, IFITM2, LYVE1, IL1RAP, PLA2G2A, PLA2G5, PCDH9, SPRY2, POSTN, KCTD12, ANG, RNASE2, RNASE4, RNASE6, ANXA1, PCSK5, PRUNE2, CD74, CSF1R, DUSP1, GPX3, KCNMB1, LCP2, GFPT2, SYNPO, A2M, EMP1, CLEC2B, COL6A3, ARL4C, RAMP1, SERPINA3, ACTN1, FOS, SERPINA1, GPR65, FBLN5, AHNAK2
LOW	D-gene vs. down-gene	KLRC3

Supplementary Table 3 | Gene list of Venn diagram. A-gene: amplification gene; D-gene: deletion gene; up-gene: up-regulated gene; down: down-regulated gene.
